# Effect of Octene Block Copolymer (OBC) and High-Density Polyethylene (HDPE) on Crystalline Morphology, Structure and Mechanical Properties of Octene Random Copolymer

**DOI:** 10.3390/polym15183655

**Published:** 2023-09-05

**Authors:** Yuan-Xia Wang, Cun-Ying Zou, Nan Bai, Qun-Feng Su, Li-Xin Song, Xian-Liang Li

**Affiliations:** 1 Polymer High Functional Film Engineering Research Center of Liaoning Province, Shenyang 110142, China; zoucunying0105@163.com (C.-Y.Z.); nanbaiasir@126.com (N.B.); m17333653774@163.com (Q.-F.S.); lixianliang007@163.com (X.-L.L.); 2 Advanced Manufacturing Institute of Polymer Industry, Shenyang University of Chemical Technology, Shenyang 110142, China; 3 College of Materials Science and Engineering, Shenyang University of Chemical Technology, Shenyang 110142, China

**Keywords:** ethylene–octene copolymer, crystallization behavior, compatibility, microstructure, mechanical properties

## Abstract

Blending octene random copolymer (ORC) with other polymers is a promising approach to improving ORC mechanical properties, such as tensile strength and elongation. In this study, octene block copolymer (OBC) with lower density than ORC and high-density polyethylene (HDPE) were used to blend with ORC. The effect of both OBC and HDPE on ORC was analyzed using scanning electron microscopy (SEM), differential scanning calorimetry (DSC), dynamic mechanical analysis (DMA) and small-angle X-ray scattering (SAXS). For ORC/OBC blends, a small amount of OBC can improve the crystallization ability of ORC. Meanwhile, for ORC/HDPE blends, the crystallization ability of ORC was significantly suppressed, attributed to good compatibility between ORC and HDPE as indicated by the homogeneous morphology and the disappearance of the α transition peak of ORC in ORC/HDPE blends. Therefore, the tensile strength and elongation of ORC/HDPE blends are significantly higher than those of ORC/OBC blends. For ORC/OBC/HDPE ternary blends, we found that when ORC:OBC:HDPE are at a ratio of 70:15:15, cocrystallization is achieved. Although HDPE improves the compatibility of ORC and OBC, the three-phase structure of the ternary blends can be observed through SAXS when HDPE and OBC exceed 30 wt%. Blending HDPE and OBC (≤30 wt%) could improve the mechanical property of ORC.

## 1. Introduction

Octene random copolymer (ORC) elastomer is a thermoplastic polyolefin elastomer synthesized by metallocene catalysis. The regular chain segments, narrow molecular weight distribution make ORC exhibit good high elasticity and plasticity [1,2,3,4,5]. Mechanical properties of elastomers can be improved by blending polymers with complementary properties [3,6,7]. Stefan et al. [8] found that blending with octene block copolymer (OBC) can optimize the application fields of olefin-based copolymers and also found that mechanical properties of semi-crystalline materials depend on both the blending components and the morphology [9,10,11,12]. The compatibility between the blends is a critical factor for properties.

OBC consists of alternating hard segments of semi-crystalline polyethylene with low ethylene content and soft segments of amorphous polyethylene with high octene content [13,14,15,16,17,18]. Compared to ORC, OBC has higher molecular chain regularity, lower glass transition temperature and higher melting temperature [19,20,21,22]. Zuo et al. [23] studied the structure and morphology during deformation of OBC and ORC with the same density and found that OBC had higher crystallization ability than ORC. Under deformation, the orientation of ORC was better than that of OBC, leading to a more effective network structure, but the tensile strength of OBC was lower.

The difference in molecular structure between ethylene–octene copolymer may lead to different effects when blended with other polymers such as ORC or OBC. Significant research has been conducted on blending ethylene–octene copolymer with high-density polyethylene (HDPE), including mechanical properties, crystallization, compatibility, morphological characteristics and rheological behavior [24,25,26,27,28,29]. Zhang et al. [30] conducted a study on blending OBC and ORC with PE. The OBC and ORC used in the study had similar crystallinity but different molecular structures. The results showed that in OBC/PE blends, a small amount of PE tended to crystallize with the hard segment in OBC, leading to thicker layers and smaller long periods. Meanwhile, in ORC/PE blends, the long period of the blend is mostly unaffected by the PE content because the PE chains in ORC tend to aggregate and form a crystal structure which is similar to pure PE. Kuang et al. [31] used HDPE to regulate the mechanical properties of OBC and found that with the increase in HDPE content, the fracture strength and tensile modulus of the blend increased. Chen et al. [32] prepared a PP/ORC/HDPE ternary blend and found that HDPE could increase the total crystallinity of the blend and improve the fracture elongation.

However, most of the ORC used in the above studies had low densities (<0.9 g/cm^3^). For the ORC with higher density than OBC, whether the compatibility between ORC and OBC or HDPE is accordance with the literature is unknown. Additionally, there are still some fundamental issues regarding the blends of OBC, ORC and HDPE that are important but lack a comprehensive understanding, such as how the synergistic influence of OBC and HDPE affects the molecular structure of ORC, as well as their impacts on crystallization dynamics and mechanical properties. We aim to study these issues. In this study, OBC with a density of 0.866 g/cm^3^ and HDPE were blended with ORC (density of 0.902 g/cm^3^) to improve the elongation and the tensile strength of ORC. The crystallization behavior of ORC/OBC, ORC/HDPE and the ORC/OBC/HDPE blends was studied. The morphology was studied by scanning electron microscopy (SEM). The packing structure and compatibility of materials were studied by small-angle X-ray scattering (SAXS) and dynamic mechanical analysis (DMA).

## 2. Experimental Part

### 2.1. Material and Sample Preparation

Two types of polyolefin elastomers and one type of high-density polyethylene were selected as the materials. The first polyolefin elastomer was ENGAGE™ 8480 (Dow Chemical Company, Midland, TX, USA), which is an ethylene–octene random copolymer (ORC) with 20 wt% octene content, a Mw of 94,500 g/mol, Mw/Mn ~2.4, a melt flow rate (MFR) of 1.0 g/10 min (230 °C, 2.160 kg) and a density of 0.902 g/cm^3^. The second polyolefin elastomer was INFUSE™ 9107 (Dow Chemical Company, Midland, TX, USA), which is an ethylene–octene block copolymer (OBC) with 12.4 wt% octene content [33], a melt flow rate (MFR) of 1.0 g/10 min (230 °C, 2.160 kg) and a density of 0.866 g/cm^3^. The selected HDPE was 52055L (Dow Chemical Company, Midland, TX, USA) with a melt flow rate (MFR) of 4.0 g/10 min (190 °C, 2.160 kg) and a density of 0.954 g/ cm^3^. 

ORC was blended with OBC and HDPE to prepare ORC/OBC, ORC/HDPE and ORC/OBC/HDPE tertiary blends using a corating twin-screw extruder with a length/diameter of 42 (WLG10, Shanghai Xinshuo Precision Machinery Co., Ltd., Shanghai, China). The extruder screw speed was 200 rpm and the feed speed was 10 r/min. The formulas of the blends are listed in Table 1. During extrusion, the temperatures of six zones were set at 170 °C, 180 °C, 180 °C, 180 °C, 180 °C and 180 °C, respectively, with a die temperature of 175 °C.

### 2.2. Thermal Analysis

A DSC (DSC3, METTLER TOLEDO, Zurich, Switzerland) equipped with liquid nitrogen cooling accessories was used to characterize the thermal behaviors of all samples. The thermal analysis was performed with a flow rate of 25 mL/min of nitrogen atmosphere. The samples (6–8 mg) were sealed in an aluminum holder and heated from ambient temperature to 200 °C at a heating rate of 30 °C/min. The temperature was maintained for 5 min to eliminate the thermal history. Then, the samples were cooled to −20 °C at a cooling rate of 10 °C/min to investigate the non-isothermal crystallization properties. To study the melting behaviors, the samples were reheated to 200 °C at the same rate after holding for 5 min at −20 °C.

### 2.3. Morphology Analysis

Scanning electron microscopy (SEM, JSM-6500, JEOL, Tokyo, Japan) was used to observe the morphology of samples. Liquid nitrogen was used to cryogenically fracture the tested samples. Then, the fractured surface of the samples was sputter-coated with Au for 2 min. Finally, the morphology of the fractured surface of samples was obtained at an accelerated voltage of 10 kV.

### 2.4. Small-Angle X-ray Scattering Measurements

The Xenocs X-ray small-angle scatter meter (Xeuss 2.0, Grenoble, France) was used to measure the microstructures and periodic structures of samples under the testing condition of copper targets. The light source wavelength was 0.154 nm, the operating voltage was 50 kV, the detector length was 1100 mm between the sample and the detector and the test time was up to 1800 s. At this distance, the effective scattering vector q is defined as q = 4πsinθ/λ, where λ is the X-ray wavelength, θ is half of the scattering angle (2θ) [11]. The two-dimensional Mar CCD detector with an active area of 165 mm in diameter recorded SAXS patterns. The collected data were revised for air background before any analysis.

### 2.5. Dynamic Mechanical Analysis

The Diamond DMA machine (Waltham, MA, USA) was used to study dynamic mechanical properties. The tested specimens were analyzed in dynamic tensile mode with a constant frequency of 1 Hz and a heating rate of 3 °C/min throughout the temperature range of −100 °C to 100 °C. The tested rectangle-shaped samples were made with dimensions of 1 mm in thickness, 50 mm in length, 10 mm in width.

### 2.6. Mechanical Testing

The Instron 3365 tensile apparatus (Instron 3365, Boston, MA, USA) was used to perform the deformation of the sample with a constant extension rate (100 mm/min) at room temperature (25 °C). The original length L_0_ between the Instron jaws was 20 mm. The samples were clamped at both ends and stretched in a symmetrical mode, where the detection spot on the sample remained fixed in space. The tested dumbbell-shaped samples were made with dimensions of 1 mm in thickness, 75 mm in length, 25 mm in neck length and 4 mm in neck width. At least five specimens were tested for each sample and average values were calculated for all mechanical properties.

## 3. Results and Discussion

### 3.1. Non-Isothermal Crystallization of the ORC/OBC, ORC/HDPE and ORC/OBC/HDPE Blends with DSC

**Figure 1:**
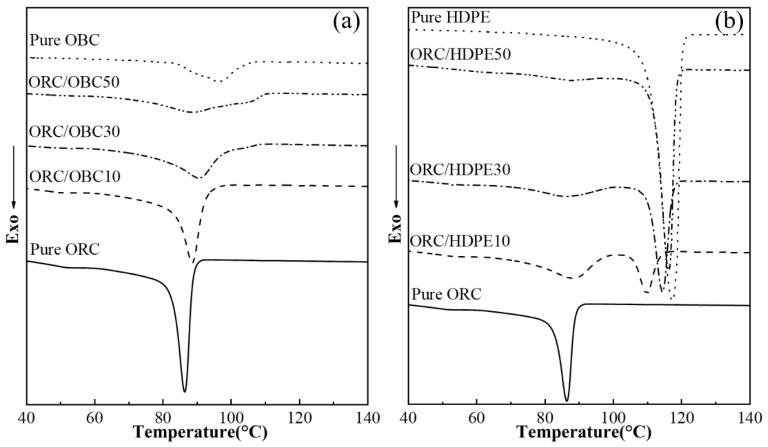
DSC cooling (10 °C/min) thermograms (**a**,**b**) of pure ORC, pure OBC, pure HDPE, ORC/OBC blends and ORC/HDPE blends.

**Figure 2:**
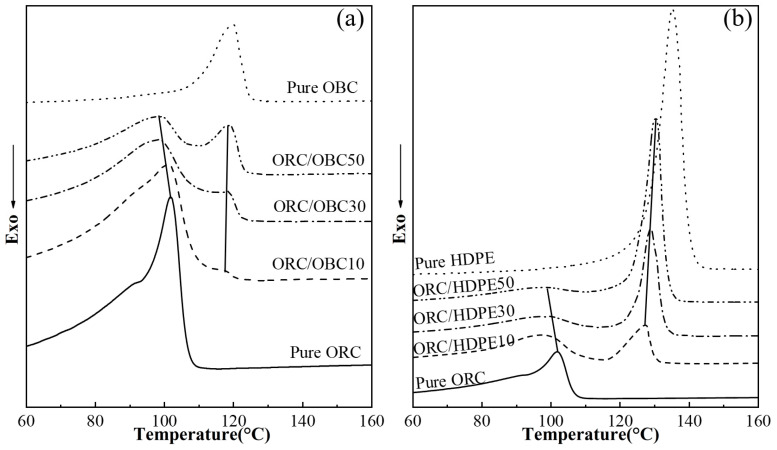
DSC the second heating (10 °C/min) thermograms (**a**,**b**) of pure ORC, pure OBC, pure HDPE, ORC/OBC blends and ORC/HDPE blends.

**Figure 3:**
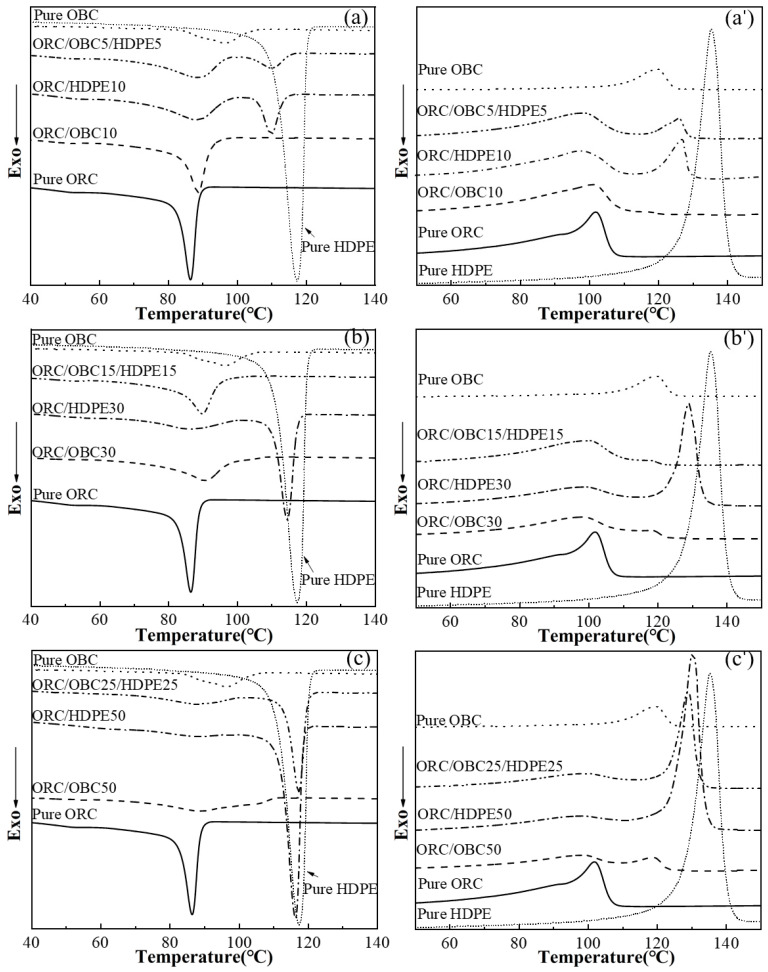
DSC cooling (10 °C/min) thermograms and the subsequent melting (10 °C/min) thermograms of pure ORC, pure OBC, pure HDPE, ORC/OBC blends, ORC/HDPE blends and ORC/OBC/HDPE blends: (**a**–**c**) cooling thermograms; (**a’**–**c’**) the subsequent melting thermograms.

ORC, OBC and HDPE are all partially crystalline polyolefins. Crystallized segments in these samples are all ethylene segments. The effects of OBC, HDPE and OBC/HDPE on the thermal properties of ORC are investigated via DSC. The cooling and second heating traces of ORC/OBC blends, ORC/HDPE blends and the ORC/OBC/HDPE blends are shown in Figure 1, Figure 2 and Figure 3, respectively. The cooling and second heating traces of pure ORC, OBC and HDPE are also presented for comparison.

As shown in Figure 1a, the crystallization peaks of pure ORC and OBC are obviously different. Pure ORC has a significant crystallization peak at 87 °C, corresponding to the crystallization of long ethylene chains in ORC. However, the ethylene chains in pure OBC have a relatively weak crystallization peak at a higher temperature (96 °C). When 10 wt% OBCs are added to ORC (see Figure 1a), the crystallization temperature of ORC increases by 2 °C, indicating that OBC improves the crystallization ability of ORC, which is different from the results in the literature that the block structure of OBC would restrict the crystallization of ORC [8]. With an increase in the amount of OBC, the crystallization temperature of ORC initially increases and then decreases, while the peak height decreases. This result can be interpreted as follows. The small amount of OBC adds more crystallization sites to the ORC due to the long PE chains in OBC, thus improving the crystallization ability of the ORC. However, when the amount of OBC in ORC/OBC is high, the geometrical constraints of OBC are dominant, resulting in weaker crystallization ability of ORC.

It has been reported that HDPE cannot accelerate the crystallization of ORC (density 0.857 g/cm^3^) [30]_._ The effects of HDPE on crystallization behavior of ORC with density of 0.902 g/cm^3^ are shown in Figure 1b. The ORC/HDPE10 blend exhibits two distinct peaks, corresponding to the crystallization of ORC (89 °C) and HDPE (110 °C), respectively. It is interesting to note that as the HDPE content in ORC increases, the crystallization peak of ORC weakens notably and the crystallization temperatures of both ORC and HDPE decrease, indicating that the ORC used in this study exhibits good compatibility with HDPE.

Figure 2a,b show the subsequent melting traces for OBC and HDPE as well as ORC/OBC and ORC/HDPE blends. Figure 2a and Table 2 show that the melting temperature of OBC in the blends remains constant (Tm_2_, located at about 120 °C), while the melting temperature of ORC decreases with a rise in OBC content. This suggests that OBC has a significant effect on ORC crystals but ORC has little effect on the crystallization of OBC. For the ORC/HDPE blends, the melting temperatures of ORC and HDPE both decrease with a rise in HDPE content (see Table 3). Since crystallized segments in these samples are all ethylene segments, the crystallinity of the samples is assessed based on the theoretical heat of melting ΔH_0_m of 293 J/g for 100% crystalline polyethylene [31]. As shown in Table 2 and Table 3, the crystallinity of the ORC/OBC blends decreases with increasing OBC content, while the crystallinity of the ORC/HDPE blends increases with increasing HDPE content.

Figure 3 shows the cooling and second heating traces of ORC/OBC/HDPE tertiary blends. Compared with pure ORC, ORC/OBC10 exhibits only one crystal peak at a higher temperature while ORC/OBC5/HDPE5 exhibits two crystalline peaks, suggesting the appearance of a phase-separation structure. It is interesting to note that the ORC/OBC15/HDPE15 blend exhibits a single crystallization peak, which indicates that ORC, OBC and HDPE cocrystallize. It can also be observed in Figure 3b’ that the ORC/OBC15/HDPE15 blend has a single melting point, while ORC/HDPE30 has two crystallization peaks (see Figure 1). Therefore, the single melting point of ORC/OBC15/HDPE15 can be interpreted as follows. Although OBC had good compatibility with HDPE, as reported in the literature [30], the ORC with higher crystallinity than OBC enters the HDPE phase, resulting in the chain segments with crystallization ability becoming shorter, while these shorter chains in HDPE crystallize with other polyethylene molecular chains in the blend. Meanwhile, OBC can improve the crystallization ability of ORC as concluded above, ultimately resulting in HDPE crystallizing together with OBC and ORC. But, for the blend with OBC and HDPE at 50 wt% in total, as shown in Figure 3c,c’, the blend exhibits two peaks again, attributed to geometrical constraints of too much OBC hindering the growth of crystals, leading to the phase separation.

The investigation mentioned above shows that OBC with lower density than ORC used in this study can improve the crystallization temperature of ORC, while the crystallization of OBC is hardly affected by ORC, evidenced by the unchanged melting point of OBC. HDPE and ORC have good compatibility, evidenced by the decreased crystalline peak at lower temperatures for both ORC and HDPE. For the ORC/OBC/HDPE blend with the ratio of ORC, OBC and HDPE of 70:15:15, ORC, OBC and HDPE can cocrystallize.

### 3.2. Morphology of Pure ORC, OBC, HDPE and the Blends

**Figure 4:**
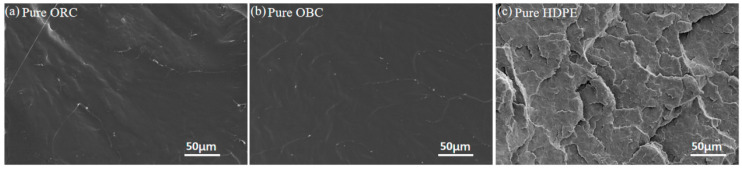
The SEM images of cryo-fracture of pure samples: (**a**) pure ORC, (**b**) pure OBC, (**c**) pure HDPE.

**Figure 5:**
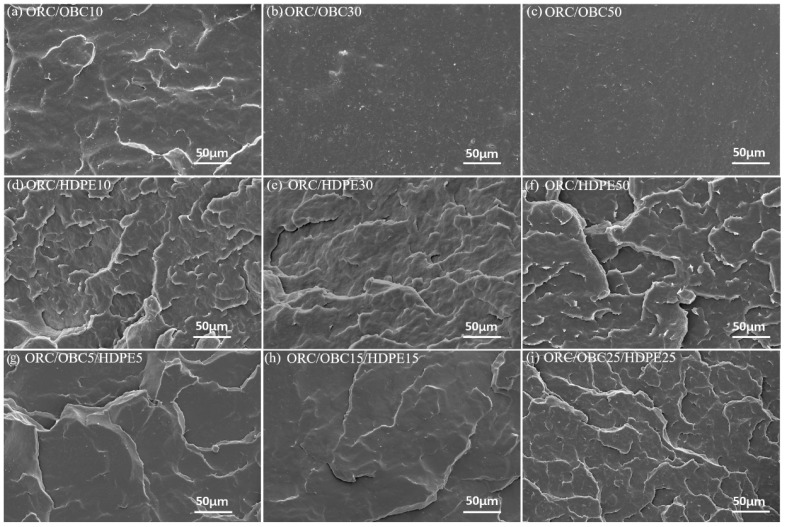
The SEM images of cryo-fracture of the blends: (**a**) ORC/OBC10; (**b**) ORC/OBC30; (**c**) ORC/OBC50; (**d**) ORC/HDPE10; (**e**) ORC/HDPE30; (**f**) ORC/HDPE50; (**g**) ORC/OBC5/HDPE5; (**h**) ORC/OBC15/HDPE15; (**i**) ORC/OBC25/HDPE25.

The SEM images of the cryo-fractured surfaces of pure samples and blends are shown in Figure 4 and Figure 5. As shown in Figure 4, ORC and OBC exhibit nearly the same ductile fracture with a smooth surface, while HDPE exhibits irregular cracks on the fractured surface and the surface is rough.

The morphology of the blends is shown in Figure 5. For the ORC/OBC10 blend, it exhibits that OBC disperses uniformly in ORC, and only a few tiny OBC particles can be observed. It is noted that with increasing content of OBC, the OBC particles became relatively obvious, which can be observed on the surface of the ORC/OBC30 blend (Figure 5b) and ORC/OBC50 blend (Figure 5c). This result suggests that despite both ORC and OBC being octene copolymers, the compatibility between ORC and OBC is limited. In contrast, the morphology of ORC/HDPE is quite different. Addition of HDPE to ORC shows a homogenous morphology, suggesting good compatibility between ORC and HDPE. Compared to ORC/OBC blends, the dispersion of ORC/HDPE seems better, implying that HDPE improves the compatibility between ORC and OBC. However, the effect of OBC and HDPE on the morphology of the fractured surfaces of the ternary blends was complicated.

### 3.3. Packing Structure of the Blends with Small-Angle X-ray Scattering

**Figure 6:**
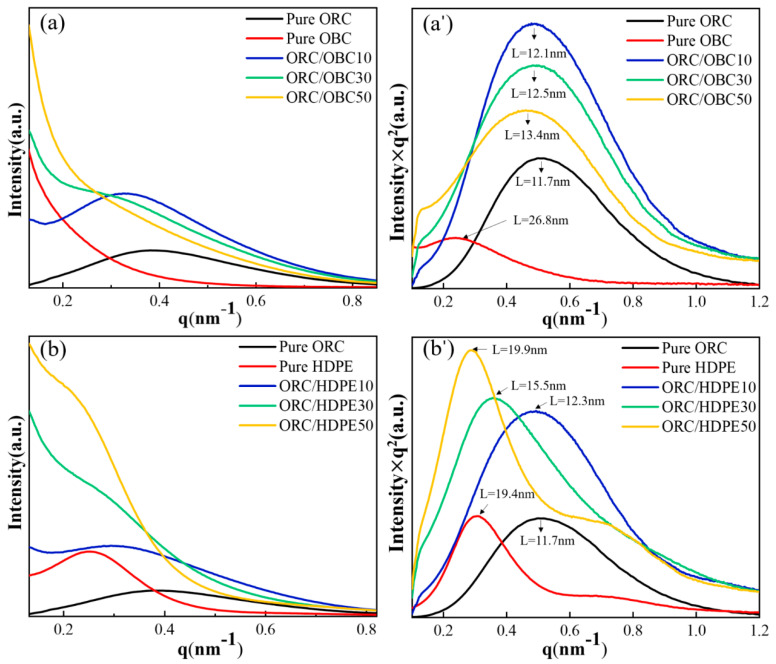
Linear and Lorentz-corrected SAXS profiles of pure ORC, pure OBC, pure HDPE, ORC/OBC blends and ORC/HDPE blends. Liner SAXS profiles: (**a**) ORC/OBC, (**b**) ORC/HDPE; Lorentz-corrected SAXS profiles: (**a’**) ORC/OBC, (**b’**) ORC/HDPE.

Small-angle X-ray scattering (SAXS) is used to further examine the microstructure of binary blends and ternary ORC blends at the nanoscale in this study. Figure 6 shows the SAXS profiles and the Lorentz-corrected profiles of these samples. The long period is used to represent the change in the average distances between adjacent crystals in a specimen, which are quantitatively evaluated from the Lorentz–SAXS [34] profiles of the sample and listed in Figure 6a’.

The octene random copolymer studied in this paper have a mixture of folded chains and micelle crystals in single crystals [8]. The crystal in HDPE is lamellar crystal. As shown in Figure 6a, pure ORC exhibits a main well-defined scattering peak around q at 0.37 nm^−1^, corresponding to the fringed micellar crystals of pure ORC. The scattering signal of pure OBC at a low q value (0.23 nm^−1^) is very high. Since the scattering intensity is proportional to the square of the density contrast, the strong scattering intensity is probably caused by the hard block segment–amorphous phase separation of OBC. Moreover, it can be observed in Lorentz-corrected profiles in Figure 6a’ that OBC also shows a well-defined scattering peak at a lower q peak position (q = 0.23 nm^−1^) than ORC, indicating the existence of polyethylene crystals. The long periods of pure ORC and pure OBC are 11.7 nm and 26.8 nm, respectively. For ORC/OBC blends, the scattering signals at a low q value (0.23 nm^−1^) are all very high, which is caused by the ORC–OBC phase separation. The original ORC peak changes to a lower peak position with the rise in OBC content, indicating that the distance between crystals is larger. It has been obtained from the DSC result that the melting point of ORC in ORC/OBC blends is lower than that of pure ORC, suggesting ORC crystals in ORC/OBC blends are smaller than in pure ORC. Therefore, the longer long period of ORC/OBC crystals results from the amorphous phase. That is to say, some OBC amorphous molecules enter the ORC amorphous phase.

The ORC/HDPE blends exhibit different trends from ORC/OBC, as shown in Figure 6b,b’. The long period of HDPE calculated from the main Lorentz–SAXS peak in Figure 6b’ is 19.4 nm. The addition of 10 wt% HDPE to ORC results in a high scattering at a low q position, which demonstrates the weak phase separation between ORC and HDPE. The long periods of ORC/OBC blends are between 12.1 and 13.4 nm, nearly the same as ORC, while the long periods of ORC/HDPE blends are between 12.3 and 19.9 nm, indicating that ORC molecules cocrystallize with HDPE.

**Figure 7:**
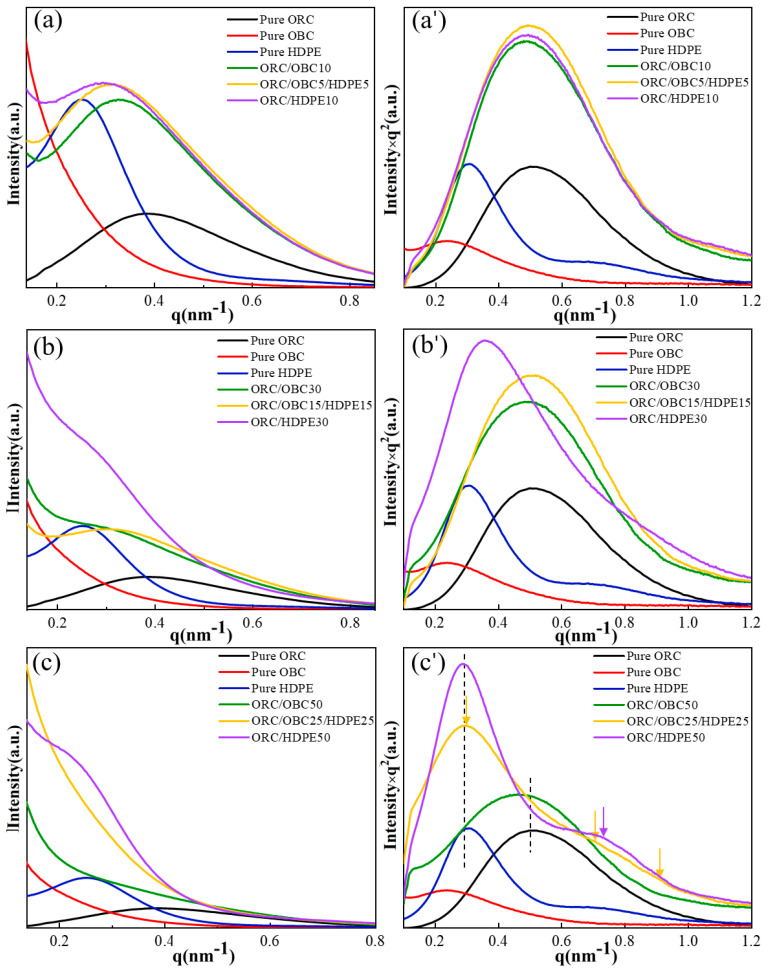
Linear and Lorentz-corrected SAXS profiles of theblends under the different conditions: (**a**–**c**) liner SAXS profiles of pure ORC, pure OBC, pure HDPE, ORC/OBC blends, ORC/HDPE blends, ORC/OBC/HDPE blends; (**a’**–**c’**) Lorentz-corrected SAXS profiles of pure ORC, pure OBC, pure HDPE, ORC/OBC blends, ORC/HDPE blends, ORC/OBC/HDPE blends.

When OBC replaced part of the HDPEs in ORC/HDPE blends, the scattering pattern was complicated. It can be seen from Figure 7a that the scattering intensity is higher at low q values for all the blends, which indicates the existence of a phase-separation structure in the blends. Moreover, compared with ORC/OBC30, a single well-defined peak is displayed at around q = 0.3 nm^−1^ for ORC/OBC15/HDPE15 and the scattering intensity at a low q value is lower than for binary blends, indicating that HDPE, OBC and ORC co-crystallize, which is in accordance with the DSC result. It is evident from Figure 7c,c’ that ORC/OBC25/HDPE25 shows three scattering peaks, which indicates that the blend has a three-phase structure. The scattering peak of the ORC/HDPE50 blend at q about 0.72 nm^−1^ is obviously larger than that of ORC/OBC25/HDPE25, indicating that the compatibility between ORC and HDPE is worse.

In summary, the phase separation between ORC and HDPE is weaker than between ORC and OBC, evidenced by scattering intensity at low q. The three-phase structure of ORC/OBC/HDPE can be observed through SAXS for the ORC/OBC25/HDPE25 blend.

### 3.4. Dynamic Mechanical Behavior

**Figure 8:**
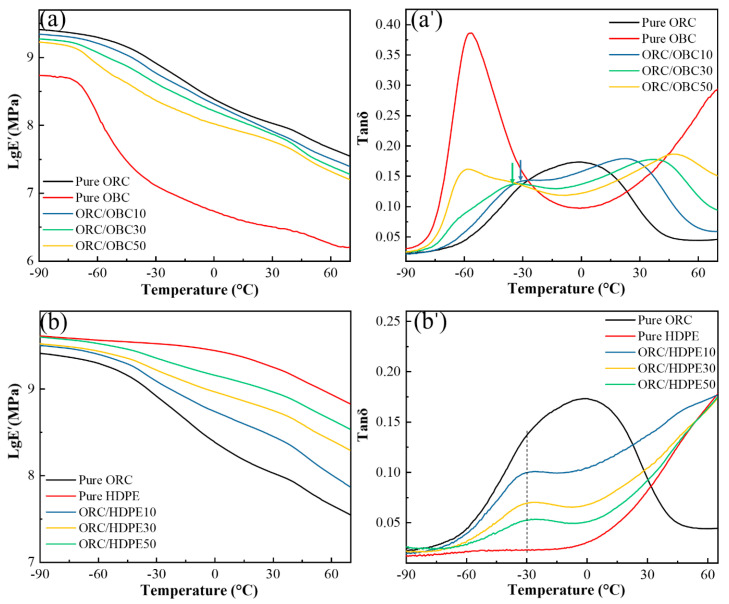
Storage modulus (**a**,**b**) and tan δ (**a’**,**b’**) of pure ORC, pure OBC, pure HDPE, ORC/OBC blends and ORC/HDPE blends.

The dynamic mechanical properties of pure ORC, pure OBC, ORC/OBC blends and ORC/HDPE blends are shown in the form of the storage modulus and loss tangent (tan δ) in Figure 8. As shown in Figure 8a, both pure ORC and pure OBC exhibit two relaxations, which can be identified as α relaxation and β relaxation (glass transition), respectively, occurring within a range of −90 °C to 70 °C. The longer plateau zone for ORC than for OBC suggests that ORC has a higher β transition temperature than OBC. Since the β relaxation is assumed to be related to the movement of the amorphous region [35,36,37,38], the higher β transition temperature of ORC resulted from the lower amount of amorphous molecular chain segments. The plateau region (β transition temperature) of the ORC/OBC blends became shorter with the rise in OBC content, indicating that OBC enters the ORC molecular chain segments, disrupting the regularity of the ORC molecular chain and increasing the amorphous region.

Figure 8a’ shows the curves of the pure ORC, pure OBC and ORC/OBC blends in terms of loss tangent (tan δ) with temperature. As shown in Figure 8a’, pure OBC shows a sharp β transition peak at −55 °C, which can be explained by the presence of a large number of amorphous components in the OBC, indicating that OBC exhibits rubber-like behavior. However, pure ORC exhibits a broad peak. The β relaxation peak of neat POE is not easily distinguishable (about −25 °C) and is covered by the α transition peak related to crystallization, which is different from the narrow peak of OBC (at about 27 °C). Compared with pure ORC, the α and β relaxations of the ORC/OBC blends are more distinct with the rise in OBC. The α transition temperature shifted towards a higher temperature direction, probably because OBC limits the movement of the ORC molecular chain segments at the phase interface. Meanwhile, the β transition temperature of ORC in the ORC/OBC blend is more pronounced than in ORC and higher than that of OBC. This can be attributed to the amorphous segments in both ORC and OBC interacting with each other and leading to a significant β transition. The blend containing 50 wt% OBC exhibits a strong β transition peak and a weak α transition peak. The β transition temperature is significantly lower and close to that of the pure OBC, indicating a more pronounced phase separation between the two components, which is in accordance with the SAXS results.

For ORC/HDPE blends (Figure 8b’), ORC in the blends exhibits a more pronounced β transition at 30 °C, which can be attributed to the incorporation of HDPE molecular chains into ORC, disrupting the regularity of ORC molecular chains and indicating pronounced compatibility between ORC and HDPE. Furthermore, the original α transition peak of ORC disappears in the blend, providing additional evidence for their compatibility.

**Figure 9:**
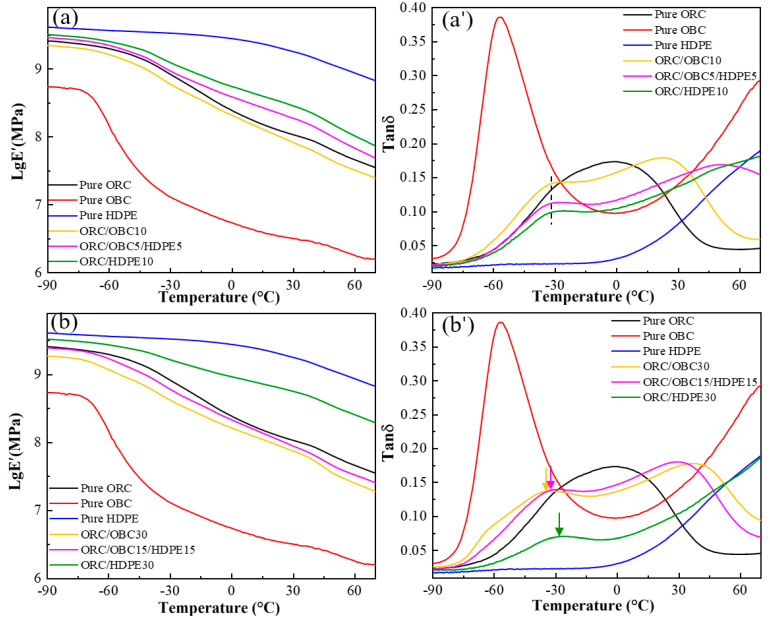

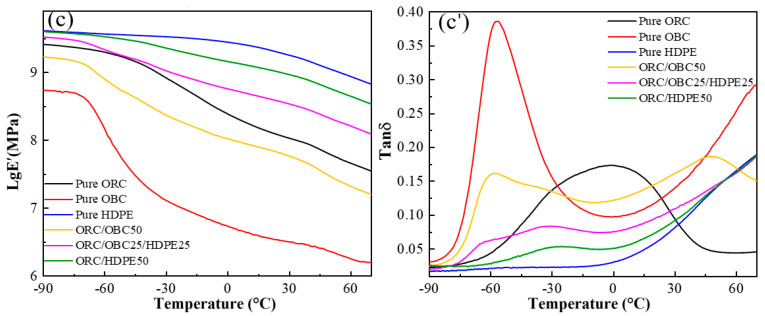
Storage modulus (**a**–**c**) and tan δ (**a’**–**c’**) of pure ORC, pure OBC, pure HDPE, ORC/OBC blends, ORC/HDPE blends and ORC/OBC/HDPE blends under the different conditions.

When HDPE is added to the ORC/OBC blends, the dynamic mechanical behavior is quite different as shown in Figure 9a,a’. Upon replacing 5 wt% of OBC with HDPE or the entire 10 wt% of OBC with HDPE, the β transition peak temperature of the blends is found to be equivalent to that of ORC/OBC10, indicating that no phase separation between HDPE and OBC is observed. It is clear that by replacing 15 wt% of OBC with HDPE, the β transition peak temperature of ORC/OBC15/HDPE15 remains almost unchanged and the α transition peak temperature is slightly lower than that of ORC/OBC30, which indicates that the compatibility of ORC, OBC and HDPE is better under this blending ratio. It should be noted that ORC/OBC25/HDPE25 exhibits two β transition peaks at around −70 °C and −45 °C (in Figure 9c,c’), which correspond to the β transitions of OBC and ORC, respectively.

The above study demonstrates that in the ORC/OBC blend with a small amount of OBC, the ORC has good compatibility with OBC using the dynamic crosslinking method, evidenced by the α and β transitions of the blend being more clearly visible. Conversely, addition of excess OBC to the blend causes a significant phase separation, as evidenced by lower glass transition temperature of OBC and ORC. HDPE exhibits good compatibility with ORC, as evidenced by the pronounced β transition temperature and lack of α transition temperature. For ternary blends, the compatibility among ORC, OBC and HDPE is greatest when the total additions of HDPE and OBC do not exceed 30 wt%, as evidenced by medium α and β transition temperatures.

### 3.5. Tensile Properties of the Blends

**Figure 10:**
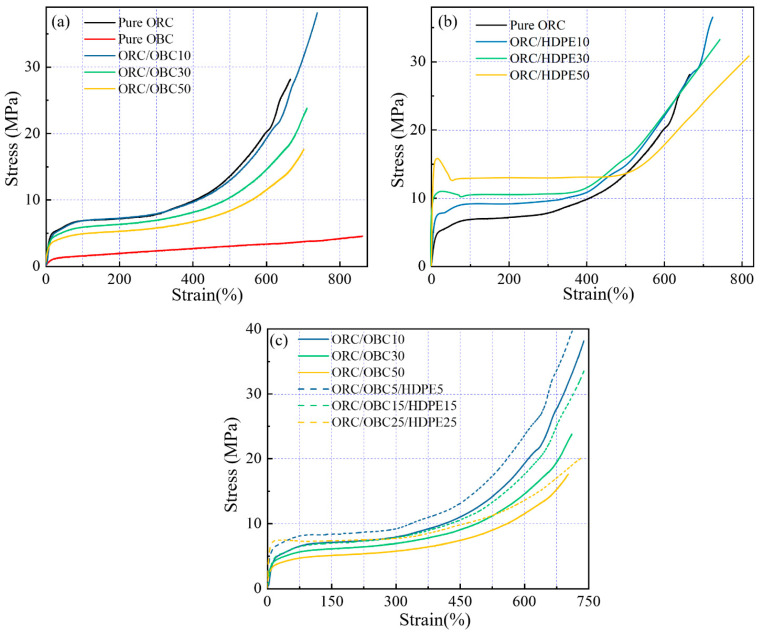
The stress–strain curves (up to break) of (**a**) pure ORC, pure OBC, ORC/OBC blends; (**b**) pure ORC and ORC/HDPE blends; (**c**) ORC/OBC blends and ORC/OBC/HDPE blends.

Figure 10 depicts the stress–strain curves of the binary and ternary blends. Table 4 shows the mechanical data of the blends. As shown in Figure 10a and Table 4, the ORC/OBC10 blend exhibits the most significant improvements in tensile strength and elongation at break compared to pure ORC. Tensile strength increases from 29.1 MPa to 38.2 MPa, while elongation at break increases from 668% to 740%. Moreover, permanent deformation decreases from 460% to 414%. This is attributed to the fact that OBC and ORC are compatible, resulting in OBC playing a positive role. With the rise in OBC, the tensile strength of ORC/OBC blends decreases due to the low stress of OBC. The elongations at break are slightly higher than those of pure ORC, demonstrating that the compatibility of OBC and ORC can play a positive role in mechanical properties of ORC only when the amount is small. It is worth noting that ORC/HDPE blends exhibit significantly higher tensile strength and higher elongation at break than ORC and ORC/OBC blends (see Figure 10b), attributed to the better compatibility of ORC and HDPE. Moreover, it can also be seen from Figure 10b that the ORC/HDPE blend displays elastomeric properties when the HDPE is 10 wt%, while it shows a yield point when the HDPE is 30 wt% and 50 wt%. This result is in accordance with the increased permanent set with the rise in HDPE.

Both pure ORC and ORC/OBC10 exhibit a stress of 6.8 MPa at 100% strain, which is attributed to the fact that ORC/OBC10 has almost the same crystallinity as pure ORC. As can be seen from Figure 10b, the tensile strength and elongation at break of the blends increase with the addition of HDPE components, which has a positive effect, but the permanent deformation increases gradually. The results can be explained as follows. The strength of HDPE is higher than that of pure ORC, and ORC enters into the HDPE phase during the stretching process and induces the surrounding matrix to produce silver grain and plastic deformation, thereby improving the toughness of ORC. However, the addition of excessive HDPE leads to a reduction in the tensile strength of the blends, which is due to the phase separation of HDPE and ORC.

As shown in Figure 10c, compared with ORC/OBC, it is evident that replacing OBC with a small amount of HDPE results in a significant increase in the permanent set, tensile strength and elongation at break of the ternary blends. Specifically, the stress at 100% and 300% strain of ORC/OBC15/HDPE15 increases by 26.4% and 25.8%, respectively. Meanwhile, the tensile strength and elongation at break are both higher than that of the ORC/OBC30 blend. It demonstrates that ORC, OBC and HDPE have good compatibility at this ratio and strong interfacial bonding ability.

## 4. Conclusions

In the present work, the effects of OBC (with lower density than ORC), HDPE and binary and tertiary blends on the crystalline behavior, structure and mechanical properties of ORC are studied. OBC used in this study improves the crystallization ability of the ORC, but ORC has a relatively small effect on the crystallization of OBC, which is evidenced by the melting temperature. HDPE weakens the crystallization ability of ORC. In the ternary blends, when the ratio of ORC, OBC and HDPE is 70:15:15, ORC, OBC and HDPE can cocrystallize.

The SEM study shows that ORC/HDPE blends exhibit homogeneous morphology, while OBC disperses comparatively worse in ORC than ORC/HDPE blends. SAXS and DMA studies show that the phase separation between ORC and HDPE is weaker than between ORC and OBC, evidenced by high scattering intensity at low q and more distinct changes in the long period in ORC/HDPE than in ORC/OBC, as well as by the pronounced β transition temperature and lack of α transition temperature from DMA. Therefore, ORC/HDPE blends exhibit significantly higher tensile strength and elongation at break than pure ORC and ORC/OBC blends. A three-phase structure can be observed through SAXS for the ORC/OBC25/HDPE25 blend. For ternary blends, the compatibility among ORC, OBC and HDPE is greatest when the total additions of HDPE and OBC do not exceed 30 wt%. A small amount of HDPE results in a significant increase in tensile strength and elongation at break of the ternary blends. Blending HDPE and OBC (≤30 wt% in total) could enhance the mechanical properties of ORC.

## Figures and Tables

**Table 1:** The formulas of the blends.

Samples	Relative Amount by Weight (wt%)		
	ORC (ENGAGE 8480)	OBC (INFUSE 9107)	HDPE (52055L)
ORC/OBC10	90	10	0
ORC/OBC30	70	30	0
ORC/OBC50	50	50	0
ORC/HDPE10	90	0	10
ORC/HDPE30	70	0	30
ORC/HDPE50	50	0	50
ORC/OBC5/HDPE5	90	5	5
ORC/OBC15/HDPE15	70	15	15
ORC/OBC25/HDPE25	50	25	25

**Table 2:** Crystallization temperature, enthalpy of fusion, melting point and crystallinity of the ORC/OBC blends.

Sample	T_C_		∆Hf (J/g)	Tm		Crystallinity (wt%)
	Tc_1_ (°C)	Tc_2_ (°C)		Tm_1_ (°C)	Tm_2_ (°C)	
ORC/OBC10	89	-	86.2	101	118	29.4
ORC/OBC30	91	106	76.7	98	118	26.2
ORC/OBC50	87	106	59.7	99	119	20.4

**Table 3:** Crystallization temperature, enthalpy of fusion, melting point and crystallinity of ORC/HDPE blends.

Sample	T_C_		∆Hf (J/g)	Tm		Crystallinity (wt%)
	Tc_1_ (°C)	Tc_2_ (°C)		Tm_1_ (°C)	Tm_2_ (°C)	
ORC/HDPE10	89	110	98.5	100	123	33.6
ORC/HDPE30	86	115	124.8	100	129	42.6
ORC/HDPE50	86	117	152.3	101	130	52.0

**Table 4:** Mechanical properties of pure ORC, pure OBC, pure HDPE, ORC/OBC blends, ORC/HDPE blends and ORC/OBC/HDPE blends.

Sample	Tensile Strength (MPa)	Elongation at Break (%)	Tensile Stress (MPa)		Permanent Set of Elongation at Break (%)
			100%	300%	
Pure ORC	29.1 ± 1.2	668 ± 20	6.8 ± 0.1	7.8 ± 0.1	460 ± 13
Pure OBC	4.5 ± 0.8	863 ± 32	1.5 ± 0.0	2.3 ± 0.1	330 ± 18
Pure HDPE	30.5 ± 0.7	1293 ± 43	15.2 ± 0.2	14.8 ± 0.0	1100 ± 22
ORC/OBC10	38.2 ± 1.1	740 ± 21	6.8 ± 0.1	7.8 ± 0.0	414 ± 11
ORC/OBC30	23.8 ± 0.6	712 ± 19	5.3 ± 0.0	6.2 ± 0.1	391 ± 9
ORC/OBC50	17.5 ± 0.5	702 ± 17	4.9 ± 0.0	5.8 ± 0.0	336 ± 12
ORC/HDPE10	36.3 ± 1.0	701 ± 20	6.8 ± 0.1	7.9 ± 0.0	474 ± 15
ORC/HDPE30	33.2 ± 0.4	746 ± 22	10.4 ± 0.1	10.7 ± 0.0	538 ± 13
ORC/HDPE50	30.7 ± 0.8	821 ± 34	12.8 ± 0.0	13.0 ± 0.2	575 ± 15
ORC/OBC5/HDPE5	40.1 ± 0.7	716 ± 22	8.2 ± 0.1	9.2 ± 0.0	455 ± 13
ORC/OBC15/HDPE15	33.6 ± 0.4	743 ± 19	6.7 ± 0.0	7.8 ± 0.0	405 ± 8
ORC/OBC25/HDPE25	20.2 ± 1.0	731 ± 24	7.3 ± 0.1	7.7 ± 0.0	538 ± 15

## Data Availability

All the data are available within this manuscript.
